# Recent Carbon Storage and Burial Exceed Historic Rates in the San Juan Bay Estuary Peri-Urban Mangrove Forests (Puerto Rico, United States)

**DOI:** 10.3389/ffgc.2021.676691

**Published:** 2021-06-07

**Authors:** Cathleen Wigand, Meagan Eagle, Benjamin L. Branoff, Stephen Balogh, Kenneth M. Miller, Rose M. Martin, Alana Hanson, Autumn J. Oczkowski, Evelyn Huertas, Joseph Loffredo, Elizabeth B. Watson

**Affiliations:** 1 U.S. EPA, Atlantic Coastal Environmental Sciences Division, Narragansett, RI, United States; 2 U.S. Geological Survey, Woods Hole Coastal and Marine Science Center, Woods Hole, MA, United States; 3 U.S. EPA, Gulf Ecosystem Measurement and Modeling Division, Gulf Breeze, FL, United States; 4 General Dynamics Information Technology, Alexandria, VA, United States; 5 Oak Ridge Institute for Science and Education Participant, Oak Ridge, TN, United States; 6 U.S. EPA, Caribbean Environmental Protection Division, Guaynabo, PR, United States; 7 Department of Biodiversity, Earth and Environmental Sciences and The Academy of Natural Sciences, Drexel University, Philadelphia, PA, United States

**Keywords:** mangrove, urbanization, tropical forest, soil carbon burial, CO_2_ emissions, carbon accumulation, carbon storage, peri-urban mangrove

## Abstract

Mangroves sequester significant quantities of organic carbon (C) because of high rates of burial in the soil and storage in biomass. We estimated mangrove forest C storage and accumulation rates in aboveground and belowground components among five sites along an urbanization gradient in the San Juan Bay Estuary, Puerto Rico. Sites included the highly urbanized and clogged Caño Martin Peña in the western half of the estuary, a series of lagoons in the center of the estuary, and a tropical forest reserve (Piñones) in the easternmost part. Radiometrically dated cores were used to determine sediment accretion and soil C storage and burial rates. Measurements of tree dendrometers coupled with allometric equations were used to estimate aboveground biomass. Estuary-wide mangrove forest C storage and accumulation rates were estimated using interpolation methods and coastal vegetation cover data. In recent decades (1970–2016), the highly urbanized Martin Peña East (MPE) site with low flushing had the highest C storage and burial rates among sites. The MPE soil carbon burial rate was over twice as great as global estimates. Mangrove forest C burial rates in recent decades were significantly greater than historic decades (1930–1970) at Cañno Martin Peña and Piñones. Although MPE and Piñones had similarly low flushing, the landscape settings (clogged canal vs forest reserve) and urbanization (high vs low) were different. Apparently, not only urbanization, but site-specific flushing patterns, landscape setting, and soil fertility affected soil C storage and burial rates. There was no difference in C burial rates between historic and recent decades at the San José and La Torrecilla lagoons. Mangrove forests had soil C burial rates ranging from 88 g m^−2^ y^−1^ at the San José lagoon to 469 g m^−2^ y^−1^ at the MPE in recent decades. Watershed anthropogenic CO_2_ emissions (1.56 million Mg C y^−1^) far exceeded the annual mangrove forest C storage rates (aboveground biomass plus soils: 17,713 Mg C y^−1^). A combination of maintaining healthy mangrove forests and reducing anthropogenic emissions might be necessary to mitigate greenhouse gas emissions in urban, tropical areas.

## INTRODUCTION

Mangrove forests globally sequester significant volumes of organic carbon (i.e., “blue carbon”) because of the long-term burial associated with their high primary production and anoxic sediments, which slow or prevent carbon (C) remineralization (Donato et al., 2011; McLeod et al., 2011; Kauffman et al., 2020). Additionally, mangrove forests are thought to store substantially more C per unit area than the world’s other major forest types (Donato et al., 2011; Alongi, 2014). In addition to providing C sequestration, tropical mangrove wetlands act as a physical barrier protecting coastal areas and communities from floods and storm surges; act as a nutrient and wastewater filter; provide habitat to fish, shellfish, and wildlife; and provide esthetic appeal and shade for people and infrastructure (Ewel et al., 1998; Gómez-Baggethun et al., 2013; Marois and Mitsch, 2015). However, the sustainability of mangrove forests is uncertain. Long-term trends in deforestation for development (e.g., aquaculture; timber) and the emerging threat of climate change, which is causing accelerated sea-level rise and increased storm intensities, are all expected to impact mangrove C sequestration (Valiela et al., 2001; Donato et al., 2011; Saintilan et al., 2020). Urbanization is also a globally pervasive stressor to tropical and subtropical mangrove forests, as it is associated with extractive pressures, increasing levels of impervious surfaces, commercial and industrial development, and geomorphic changes (Bosire et al., 2014; Lugo et al., 2014; Ochoa-Gómez et al., 2019). Human alterations of the landscape and the natural geomorphology (e.g., canalization; dredging) can increase the exposure of mangrove forests to wastewater, nutrients, and toxicants (Bosire et al., 2014; Lugo et al., 2014; Branoff, 2020b). In some peri-urban (i.e., adjacent to a city) mangrove systems (e.g., Mombasa and Kenya) extractive pressures (i.e., illegal wood extraction) are still a dominant cause for degradation (Bosire et al., 2014), while in other mangrove wetlands located next to urban areas (e.g., Puerto Rico), legal protections have reduced extractive pressures and allowed for mangrove expansion (Martinuzzi et al., 2009).

Now, in the Anthropocene Epoch, with its associated stressors such as global climate change, it is more important than ever to examine the effects of local urbanization on C sequestration in coastal mangroves (Bosire et al., 2014; Ochoa-Gómez et al., 2019). The global trends in peri-urban mangrove forests show nitrogen (N) enrichment due to human watershed activities and significantly lower C to N ratios in mangrove biomass (Branoff, 2017, 2019). In a global review of 66 dated mangrove cores, Pérez et al. (2018) reported that mangrove ecosystems receiving domestic or aquaculture effluents had sediment accretion and organic carbon accumulation rates twofold and fourfold, respectively, higher than mangrove systems in conserved sedimentary environments. Mangrove systems may play an important role in effectively sequestering nutrient inputs and carbon in urbanized coastal areas.

**FIGURE 1 |:**
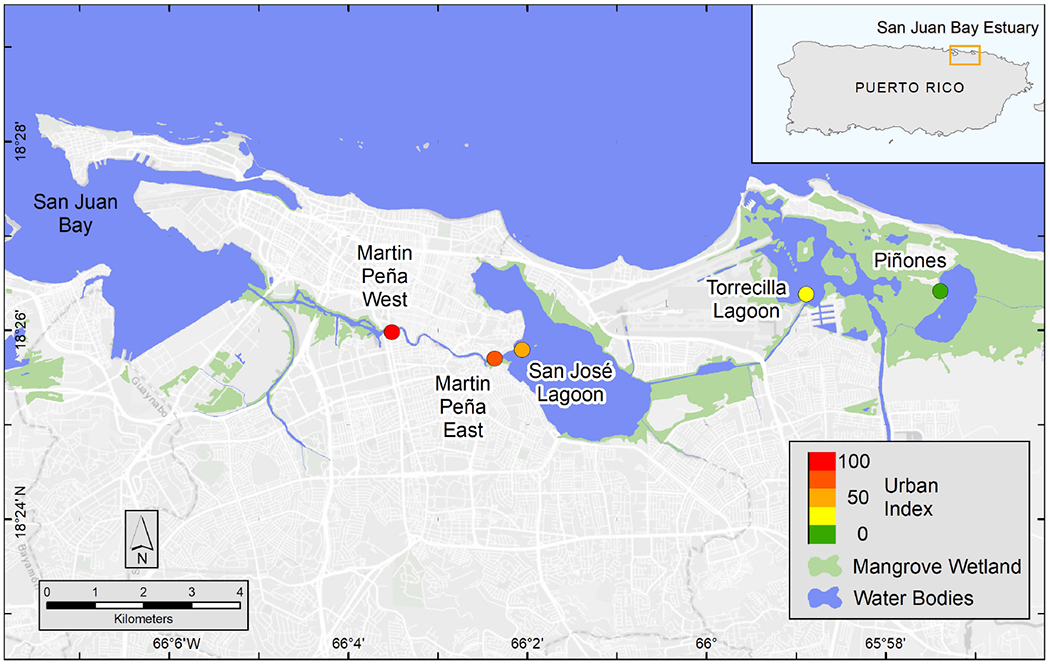
Map of San Juan Bay Estuary mangrove study sites and urbanization score (colored circles). The Urban Index was scored on a scale from 1 to 100 and based on surrounding coverage of mangrove wetland, non-mangrove vegetation and open water, urban land, population density, and road density, which were determined in a 500 m buffer from the approximate center of the mangrove site. National Wetland Inventory data were used to map mangrove wetlands, which included estuarine, intertidal, forest and scrub shrub, broad-leaved evergreen habitats (U.S. Fish and Wildlife Service, 2018).

As the economy of Puerto Rico shifted from agriculture toward industry in the 1940s, people abandoned rural areas and migrated to the coastal lowlands, such as the San Juan Metropolitan Area where commerce, industry, and transportation were rapidly developing (Martinuzzi et al., 2009). Today, the economy is shifting away from light industry, which was mainly pharmaceutical production to commercial services (Feliciano, 2018). Population has decreased in San Juan since the early 2000s. Despite the population decline, the San Juan Bay Estuary (SJBE) watershed currently has the densest human population on the island, ~1,850 people km^−2^, primarily located in the urban municipalities (i.e., San Juan, Carolina, and Bayamón) associated with the city of San Juan, Puerto Rico (U.S. Census Bureau, 2017). The mangrove forests in Puerto Rico’s SJBE are located along a well-established, west to east urbanization gradient (Oczkowski et al., 2020a,b; Figure 1). The western canals of the densely populated SJBE are heavily contaminated by sewage with fecal coliform concentrations exceeding 2 × 10^6^ cfu 100 ml^−1^ (Puerto Rico health standards are 200 cfu 100 ml^−1^; Army Corps, 2016) and are exposed to large amounts of urban runoff including wastewater and pump station stormwater discharges. In contrast, the easternmost part of the gradient consists primarily of a forest reserve with the least disturbed habitat in the SJBE (Pérez-Villalona et al., 2015; Oczkowski et al., 2020a,b).

Tidal connectivity, flushing, temperature, salinity, and nutrient inputs are known to influence primary productivity and C sequestration in mangroves (Krauss et al., 2006; Lugo and Medina, 2014; Lugo et al., 2014). These parameters in the urbanized SJBE have been heavily altered by bridge building, canalization, dredging, filling, and extensive shoreline development for more than two centuries (Ellis, 1976; Cerco et al., 2003; Branoff, 2020a). At the western end of the SJBE, repeated dredging of a portion of the Caño Martin Peña since the 1920s allowed for improved exchange between San Juan Bay and the western end of the canal, but the clogged eastern end of the canal [Martin Peña East (MPE)] prevents tidal connectivity into the eastern lagoonal systems of the SJBE. The dredging of the Suárez Canal in the 1960s somewhat improved tidal exchange between San José Lagoon (SJ) and La Torrecilla Lagoon (Torr), and the dredging of La Torrecilla Inlet to the sea in the late 1960s and early 1970s enhanced the tidal exchange. At the easternmost end of the SJBE, the mature Piñones Forest (Pin) is a relatively isolated inland lagoon displaying a weak hydrologic connection with the rest of the estuary and only limited exchange with the sea (Ellis, 1976; Lugo et al., 2011).

Mangrove forests in the SJBE were composed of Red (*Rhizophora mangle* L), White [*Laguncularia racemosa* (L.) C.F. Gaertn], Black [*Avicennia germinans* (L.) L.], and Button [*Conocarpus erectus* (L.)] mangrove species, and numbered over five million trees (with diameter at breast height ≥2.54 cm) in 2011 (Brandeis et al., 2014). Mangrove C storage and accumulation rates were determined from tree density and above and belowground biomass, which was estimated from allometric equations and above/belowground ratios. Carbon accumulation rates of mangroves in the SJBE in 2011 were estimated to be ~11,262 Mg y^−1^ (Brandeis et al., 2014). In the present study we update and expand on the 2011 estimates of C storage rates in the urban SJBE mangroves, previously based only on biomass estimates, by examining the mangrove aboveground production as well as the C burial in the soils at five mangrove forest sites spanning the SJBE from west to east.

The study aims to elucidate the historic (1930–1970) and recent (1970–2016) decadal mangrove forest C storage and burial rates along an urbanization gradient in the SJBE. Using radiometrically (^210^Pb, ^137^Cs) dated cores from the mangrove study sites we account for recent and historic decadal soil accretion rates. We estimate the mangrove forest C sequestration (aboveground plus soils) at the scale of the local mangrove forest and the entire estuary. For comparative purposes we examine the magnitude of the estuary-wide mangrove forest C sequestration relative to watershed CO_2_ emission rates due to human activities (vehicular, residential, industrial, and commercial use). Using percent C, soil bulk density, and accretion rates we describe C storage and burial rates. We examine C stable isotope and C/N ratios in the soils to identify dominant sources of C. We hypothesize that accretion and carbon burial rates (CBRs) are greater in more urban watersheds and in more recent decades (1970–2016) compared with earlier decades (1930–1970), attributable to increases in human activities (e.g., wastewater inputs; filling in with debris) and alterations associated with rapid urbanization (e.g., dredging activities; damming of rivers). We discuss how site-specific differences in landscape setting, soil fertility, and flushing patterns may affect C storage and accumulation rates in the mangrove forests.

## MATERIALS AND METHODS

### Site Description

The coastal extent of the tropical, urban SJBE is over 21,658 ha and includes highly developed and dredged areas, clogged canals, lagoons, and a relatively undeveloped mangrove forest reserve (Cerco et al., 2003; Brandeis et al., 2014; Oczkowski et al., 2020b). The locations of the mangrove stands we sampled included the Caño Martin Peña in the western half of the estuary, a series of lagoons in the center of the estuary, and the easternmost part of the estuary, that is the largest remaining contiguous mangrove forest in Puerto Rico (Figure 1; see Oczkowski et al., 2020a for a more detailed description of the study sites). We classify the relative flushing, landscape setting, and degree of urbanization among the different sites based on previous reports which included water level observations sampled throughout 2016–2018, assessments of the landscape geomorphology, and detailed analyses of hydrology and watershed land use (Branoff, 2020b; Martin et al., 2020). The Urban Index was previously reported for the SJBE mangrove sites and was derived from five spatial variables: (1) surrounding coverage of mangrove vegetation, (2) non-mangrove vegetation and open water, (3) urban land, (4) population density, and (5) road density, which were calculated within a 500 m buffer from the approximate center of the mangrove site (Martin et al., 2020; Table 1). The index was a relative metric of the level of urbanization with a score of 100 being the most disturbed and 1 being the least disturbed (Branoff, 2019, 2020b). The Caño Martin Peña, located in the western half of the estuary, was associated with high population density, impervious surfaces, commercial and industrial development, and had a high Urban Index (88–100), while the expansive Pin in the easternmost part was scored 1 (Martin et al., 2020; Table 1).

### Core Collections and Analyses

Mangrove sediment cores, two from each site, ~1 m apart, were collected in March 2016 in the Martin Peña West (MPW), MPE, SJ, Torr, and Pin with a Russian peat sampler to a maximum depth of 50 cm depending upon ability to penetrate coarse mangrove rhizomes. Core depths ranged from 37–50 cm. One core collected from MPW was damaged during air transport to the United States mainland and was not radiometrically dated. The other nine cores were sliced in one cm increments at the surface (0–3 cm) and then every 2 cm to the bottom of the core (unless otherwise indicated). Soil subsamples were used for radiometric dating, stable isotope, percent C, N, dry bulk density (DBD), and sediment accretion analyses as described below. However, only one of the two replicates from the SJ was processed for DBD due to human error, so DBD values from one SJ core were used in calculations of C storage and burial rates for both replicates from that site.

### Processing for Bulk Density, C Stable Isotopes, and Percent C, N

Soil subsamples were dried at 60°C for at least 48 h. The soils were not sieved so the belowground samples included live and decomposing roots. One portion of the dried soils was used to determine DBD (g cm^−3^) and the other portion was ground to a fine powder using a mortar and pestle. The dried, ground material was used for determining C stable isotopes and percent C. Samples were fumigated prior to analyses with 12 M HCl following the method of Harris et al. (2001) to remove carbonates. The C isotope compositions were determined using an Elementar Vario Micro elemental analyzer connected to a continuous flow Isoprime 100 isotope ratio mass spectrometer (IRMS) (Elementar Americas, Mt. Laurel, NJ, United States). Replicate analyses of isotopic standard reference materials USGS 40 (δ^13^C = −26.3‰) and USGS 41 (δ^13^C = 37.63‰) were used to normalize isotopic values of working standards to Vienna Pee Dee Belemnite (δ^13^C) scales (Paul et al., 2007).

Stable isotope values are expressed in δ notation following the formula δX (‰) = [(Rsample/Rstandard) − 1] × 10^3^, where X is the less common isotope and R is ratio of the less common to more common isotope (^13^C/^12^C). Working standards were analyzed every 24 samples to monitor instrument performance and check data normalization. The precision of the laboratory standards was better than ±0.3‰ for δ^13^C. The % C and % N were calculated by comparing the peak area of the unknown sample to a standard curve of peak area vs the C or N content of a known standard.

Potential organic matter sources were examined by plotting sediment molar C/N ratio vs sediment C stable isotopes in core sediments relative to potential organic matter sources. The source δ^13^C and C/N ratios associated with seagrass, marine algae, particulate organic matter (POM), and mangrove plant matter was based on literature values (Khan et al., 2019; Branoff, 2019; Oczkowski et al., unpublished data). Ranges of C/N ratios and δ^13^C for mangrove-derived source material was separated into roots and leaves (green only), and further characterized by dominant species, Red (*R. mangle* L.), White [*L, racemosa* (L.) C.F. Gaertn.], and Black [*A. germinans* (L.) L.] species (based on Khan et al., 2019; Branoff, 2019).

### Radiometric Dating

Gamma analysis was performed on 10–20 samples that spanned each sediment core. One to ten grams of homogenized sediment were sealed for 3 weeks and counted on a planar-type gamma counter for 24–48 h to measure ^137^Cs, ^210^Pb, and ^226^Ra at 661.6, 46.5, and 352 KeV energies respectively (Canberra Inc., United States). Activities of ^137^Cs and ^210^Pb were decay corrected to time of collection; suppression of low energy peaks by self-adsorption was corrected according to Cutshall et al. (1983). Age models were developed using Plum (Aquino-López et al., 2018; Blaauw et al., 2020) version 0.1.4 in R version 4.0.0 (R Core Team, 2020). Plum is an age-depth model that utilizes ^210^Pb and is based on the same Bayesian chronology statistical treatment as Bacon, a widely used model with ^14^C ages (Blaauw and Christen, 2011). Plum uses distributions of prior environmental parameters that impact the ^210^Pb profile, including ^210^Pb deposition rates, supported ^210^Pb (i.e., ^226^Ra) and accretion rates, with posterior distributions providing realistic uncertainty estimates. A major benefit of Plum over the commonly used analytical solution to the continuous rate of supply model (Appleby and Oldfield, 1978) is that chronologies can be calculated even if radioisotopes have not been analyzed for the entire core. Furthermore, this model yields more than one accretion rate estimate, unlike the constant initial concentration model (Goldberg, 1963) which is normally used for cores with discontinuous ^210^PB profiles. Total ^210^Pb data were input into Plum, with supported ^210^Pb (i.e., ^226^Ra) estimated within the model framework from the deepest samples. We broadened the priors from default settings within Plum. We simulated means (50%) and the lower (2.5%) and upper confidence limits (97.5%) for each 1 cm depth in the sediment cores. We report means and 95% confidence intervals in the age-depth profiles for each core. Sediment accretion rates (SAR) were obtained from each chronology using the “accrate depth” function in Plum at 1 cm depth intervals. Age-depth models such as these constrain accretion relative to the sediment surface at the time of collection; thus, interpretations of absolute elevation gain within a fixed framework, such as sea level, is not possible without further measurements, such as from sediment elevation tables (Cahoon et al., 2002). However, these age-depth models are useful for constraining potential maximal elevation changes.

### Calculations of Estuary-Wide C Storage and Accumulation Rates in Mangrove Forest

To estimate estuary-wide mangrove forest C storage and accumulation rates, we interpolated aboveground biomass and belowground soil measurements onto a grid of 2 × 2 m mangrove areas represented by the “estuarine forested wetlands” class in the NOAA C-CAP dataset (Office for Coastal Management, 2017). This estimate combined both short-term (years to decades) C sequestered in aboveground biomass with long-term (decades to century) C sequestered in the soils of mangrove forest. Belowground soil measurements were estimated from the nine cores collected in the present study and aboveground biomass measurements were from Branoff and Martinuzzi (2020) for C storage and Branoff (2020a) for C accumulation, assuming 44% C content in aboveground biomass (Rodrigues et al., 2015). Aboveground biomass measurements were taken prior to the hurricane season in 2017 (Branoff, 2020a). Interpolation was computed as an inverse distance weighting (Shepard, 1968) through the *idw* function of the phylin package in R (Tarroso et al., 2015), in which values from all known locations were incorporated into the interpolation but the geographically closest known measurements were given higher weight. To incorporate the known variability in the field measurements, interpolation was bootstrapped for 100 iterations, with known values randomly drawn from right skewed normal distributions of the same mean, standard deviation, and 95% confidence intervals as the measured values. Distributions were simulated through the *rsnorm* function of the fGarch package in R (Wuertz et al., 2020). Watershed-wide estimates were then calculated by summing the mean values for each mangrove area and each C component (aboveground, recent soils, and historic soils) from the bootstrapped iterations, with mean and standard errors of these iterations reported.

### Calculation of Emissions by People in the SJBE Watersheds

To estimate the local CO_2_ emissions by people and their activities, we characterized the land cover and estimated the human population, households, and vehicles owned in the SJBE watershed in ArcGIS using landscape datasets (Hamilton and Casey, 2016; Office for Coastal Management, 2017) and U.S. Census Bureau (2017). These land class areas and demographic data were used to estimate annual CO_2_ emissions from industrial ecology methods (Ngo and Pataki, 2008; Kennedy et al., 2010). Specifically, we calculated per-household CO_2_ emissions by multiplying the number of occupied households in the watershed by residential, commercial, and industrial electricity consumption factors derived from energy consumption data reported in the published literature (Supplementary Table 1a). For personal automobile emissions, we multiplied the number of vehicles in the watershed by the average annual vehicle-miles traveled by residents on the island and the average fuel efficiency (Supplementary Table 1a). We tallied electricity and gasoline consumption for the watershed and converted to CO_2_ emissions by multiplying by EPA emissions factors (U.S. Environmental Protection Agency, 2014). We report the magnitude of the watershed anthropogenic CO_2_ emissions relative to the magnitude of the mangrove forest C sequestration rates (aboveground plus soils) for comparative purposes and to inform management efforts assessing anthropogenic impacts and mangrove forest blue carbon value (McLeod et al., 2011). We did not measure actual mangrove atmospheric/canopy fluxes.

### Site and Time Period Statistical Comparisons

Mean SAR, DBD, % C, and δ^13^C were calculated within two identified time periods: recent decades, approximately the 1970s to present-day (i.e., 2016, date of collection) and historic decades (1930s to 1970s). The historic decades represent a period of mangrove recovery following over a century of intense agricultural activity (e.g., conversion of lands to sugar cane fields and pastures) in the 1800s (Martinuzzi et al., 2009). The recent decades represent a period characterized by rapid urbanization with watershed land development and increases in population, but also by legal protection of all mangroves on the island in 1972 (Martinuzzi et al., 2009).

Defined time periods were determined from the simulated means in the age-depth models generated by radiometric dating and Plum models. We used a bootstrap approach to generate means and 95% confidence bounds for each parameter within sites, cores, and specific time periods. The bootstrap analysis generated 1,000 sets of randomly selected data from the reported values for the specific time period in a given core (Efron and Tibshirani, 1993). The mean of those 1,000 values was used as the overall mean estimate for the parameter, and the 2.5th and 97.5th percentiles of those 1,000 values were the upper and lower confidence bounds.

Using the SAR, DBD, and % C parameters we calculated mangrove accretion rates (MARs), CBRs, C density, and C storage for each time period (see Supplementary Table 2 for formulas). To better encompass the propagation of variability resulting from each individual measurement included in the calculation of these parameters, the bootstrap generated 1,000 sets of randomly selected pairings of the variables going into the calculation. For example, the C density calculation was based on randomly selecting a DBD and a % C value from combined cores from a specific site in a specific time period, but not necessarily with each value coming from the same depth. The means and 2.5th and 97.5th percentiles of the 1,000 sets of calculated values would be the overall mean and confidence bounds, as with the other parameters.

For examination of spatial comparisons among mangrove sites the 1,000 bootstrap estimates from both core replicates from a specific site were combined to calculate site specific means and bounds. The mean of those 2,000 values was used as the overall mean estimate for the parameter, and the 2.5th and 97.5th percentiles of those 2,000 values were the upper and lower confidence bounds. The site-specific bounds generated by combining the bootstrap estimates tended to be wider than the individual core-specific bounds when the variability among the two cores was large. However, comparisons made based on these site bounds will enable more meaningful evaluations of spatial differences.

For temporal comparisons, because core variability can mask changes over time within individual core locations, we examined whether significant differences existed between recent vs historic time period means using the bounds generated based on each of the 1,000 core-specific bootstrap estimates. For example, the historic period mean and bounds generated from the 1,000 bootstrap runs for core PIN1 would be compared to the recent period mean and bounds generated from PIN1, while a separate comparison would be done using the PIN2 data and bootstrap bounds.

For both the spatial and temporal comparisons, significant differences were evaluated based on whether bootstrap bounds (2.5th and 97.5th percentiles) overlapped. For C storage comparisons between recent (1970–2016) and historic time periods (1930–1970), values were normalized to account for the difference in range between time periods (46 vs 40 years); however, the statistical differences in C storage results between time periods did not change whether the data were normalized or not.

## RESULTS

### Soil C Burial and Storage Rates

**FIGURE 2 |:**
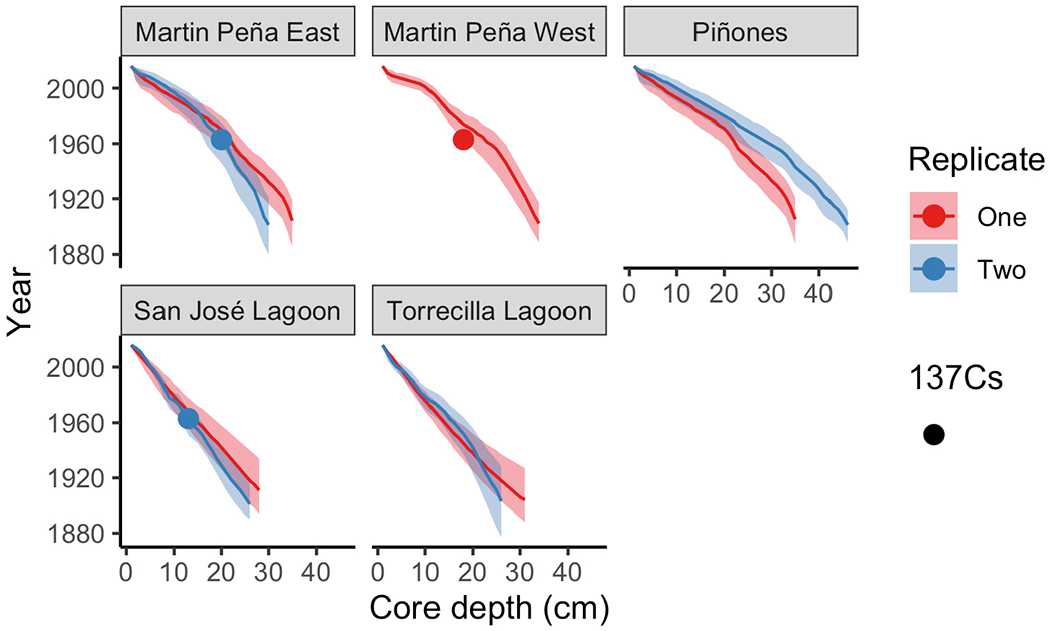
Core age-depth profiles using the Plum model (Aquino-López et al., 2018) estimated sediment year with depth in each core. Means (solid line) and 95% confidence intervals are depicted for each core. Cores with detectable ^137^Cs peaks are indicated with circles.

We report age means and 95% confidence intervals in the age-depth profiles for each core and use these estimates to calculate SAR (Figure 2). The raw radiometric and associated C, N data from this study are available in a USGS data release (Eagle et al., 2021). The site mean SAR ranged from 1.96 mm y^−1^ at SJ to 3.76 mm y^−1^ at Pin in historic decades, and there was no significant difference among sites. Piñones (5.52 mm y^−1^), MPE (4.91 mm y^−1^), and Martin PeñaWest (5.47 mm y^−1^) had similar SAR site means in recent decades, and these were significantly greater than the SARs at La Torrecilla (2.68 mm y^−1^) and San José (2.02 mm y^−1^) lagoons (Table 2 and Supplementary Table 3). SARs in the Caño Martin Peña and Piñones cores were significantly greater in recent than historic times. In the lagoonal cores, SJ1 and Torr1, the recent SARs were less than the historic SARs (Supplementary Table 4).

**FIGURE 3 |:**
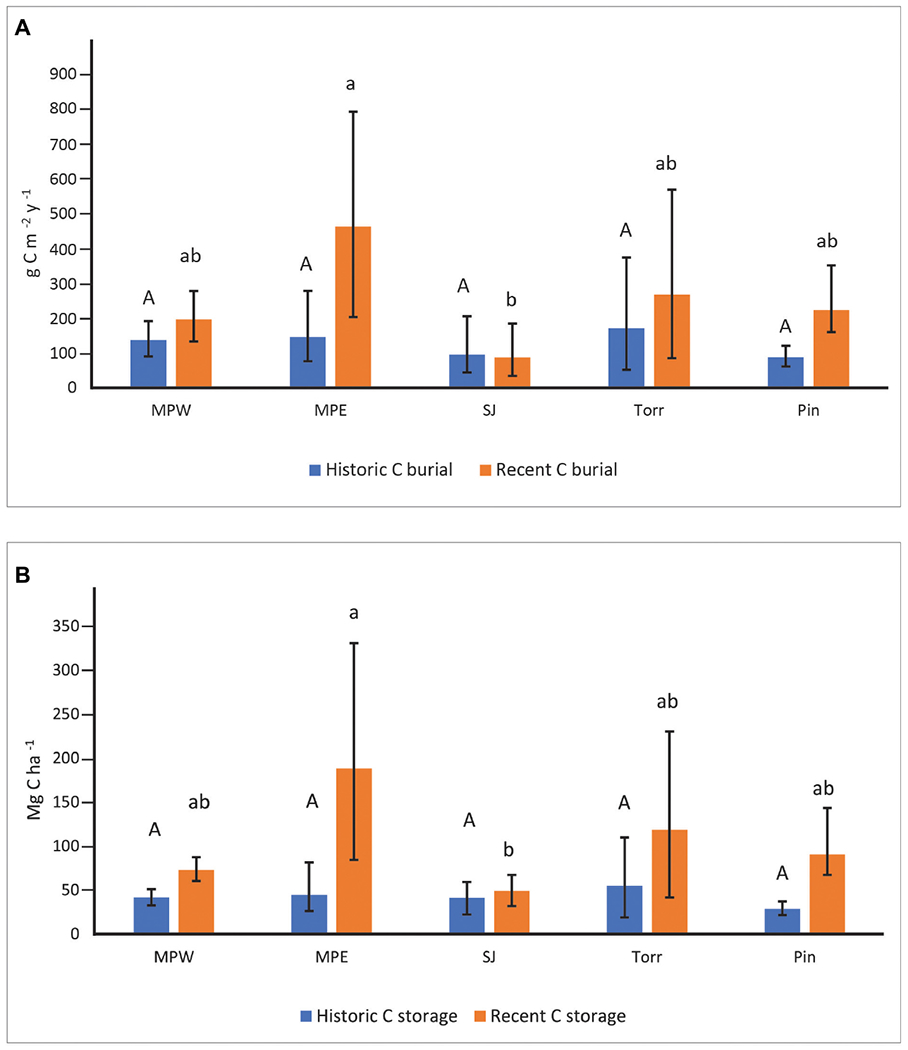
Soil carbon burial rates **(A)** and soil carbon storage **(B)** at the mangrove sites in recent (1970–2016) and historic (1930–1970) decades. Upper-case letters were used to describe site comparisons within historic decades and lower-case letters to describe site comparisons within recent decades. Mangrove sites that do not share letters within a specific time period had significantly different (*P* < 0.05) values. Statistical site differences within time periods were based on whether bootstrapped bounds (i.e., 2.5th and 97.5th percentiles) overlapped. Parameter means, lower, and upper confidence bounds were generated on bootstrap runs (1,000 bootstrap values per core; combined for a total of 2,000 values for sites with two replicates). Sites listed from high to low urbanization index (see Table 1 for site abbreviations).

Soil C burial rates were not significantly different among sites in historic decades (Figure 3A). In recent decades, the highly developed MPE with low flushing had the highest soil C burial rates (mean: 469 g m^−2^ y^−1^; 95% bounds: 205–803 g m^−2^ y^−1^). These rates were significantly greater than C burial rates at San José, but not statistically different than La Torrecilla, Piñones, or MPW (Figure 3A). Although the SJ cores had significantly higher % C than all the other sites in historic and recent decades (Supplementary Table 5), the sediments were characterized by low DBDs and low SARs that resulted in generally low CBRs (Figure 3A and Supplementary Tables 4, 5). Soil C burial rates in recent decades were significantly greater than historic decades for each core within MPE and Piñones. Although Piñones and MPE had similarly low flushing, the landscape settings (forest reserve vs clogged canal) and urbanization (lowest vs high) were not similar (Table 1), suggesting the importance of flushing in affecting C burial. For all other sites, there was no statistical difference in CBR between historic and recent decades.

Soil C storage ranged from 28.71–55.19 Mg ha^−1^ among mangrove forest sites in historic decades, and there was no significant difference among forests (Figure 3B). In recent decades, MPE had the highest soil C storage (187.90 Mg ha^−1^), but it was not significantly different than soil C storage in La Torrecilla (118.56 Mg ha^−1^), Piñones (90.50 Mg ha^−1^), or MPW (72.97 Mg ha^−1^) (Figure 3B). Soil C storage at MPE was significantly greater than C storage at SJ (48.85 Mg ha^−1^). Greater soil C storage in recent decades as compared to historic decades was evident in all SJBE mangrove cores except three lagoonal cores: SJ1, SJ2, and Torr1. Having similar DBD and % C in both historic and recent time periods (Supplementary Table 5), resulted in no significant difference in C storage between time periods in these three lagoonal cores. Cores collected from Torr had the highest variability in DBD, with the DBD of Torr2 about 4–8 times greater than Torr1 (Supplementary Table 5).

The MAR is dependent upon the DBD and SAR of the soil, and in historic and recent decades the SJ (historic: 309.77 g m^−2^ y^−1^; recent: 291.80 g m^−2^ y^−1^) had significantly lower MAR than the Caño Martin Peña sites (historic: 1,531.33 g m^−2^ y^−1^; recent: 2,855.81 g m^−2^ y^−1^) and Pin (historic: 1,926.93 g m^−2^ y^−1^; recent: 3,770.30 g m^−2^ y^−1^) (Table 2 and Supplementary Table 3). There were no differences in MAR between historic and recent decades in most SJBE cores, except for cores MPE2 and Pin 1, in which recent MAR was significantly greater than historic MAR. Similar to MAR, there were no differences in C density between historic and recent decades in most SJBE cores, except for cores MPE1, Pin 1, and Pin2, in which recent C density was significantly greater than historic C density (Supplementary Table 4). In historic decades the Caño Martin Peña sites (0.053 g cm^−3^) had significantly higher C density than Piñones (0.024 g cm^−3^), while in recent decades the MPE (0.096 g cm^−3^) had significantly greater C density than MPW (0.036 g cm^−3^). All other sites had similar C densities as the Caño Martin Peña in recent decades (Table 2 and Supplementary Table 3).

### Carbon Sources

**FIGURE 4 |:**
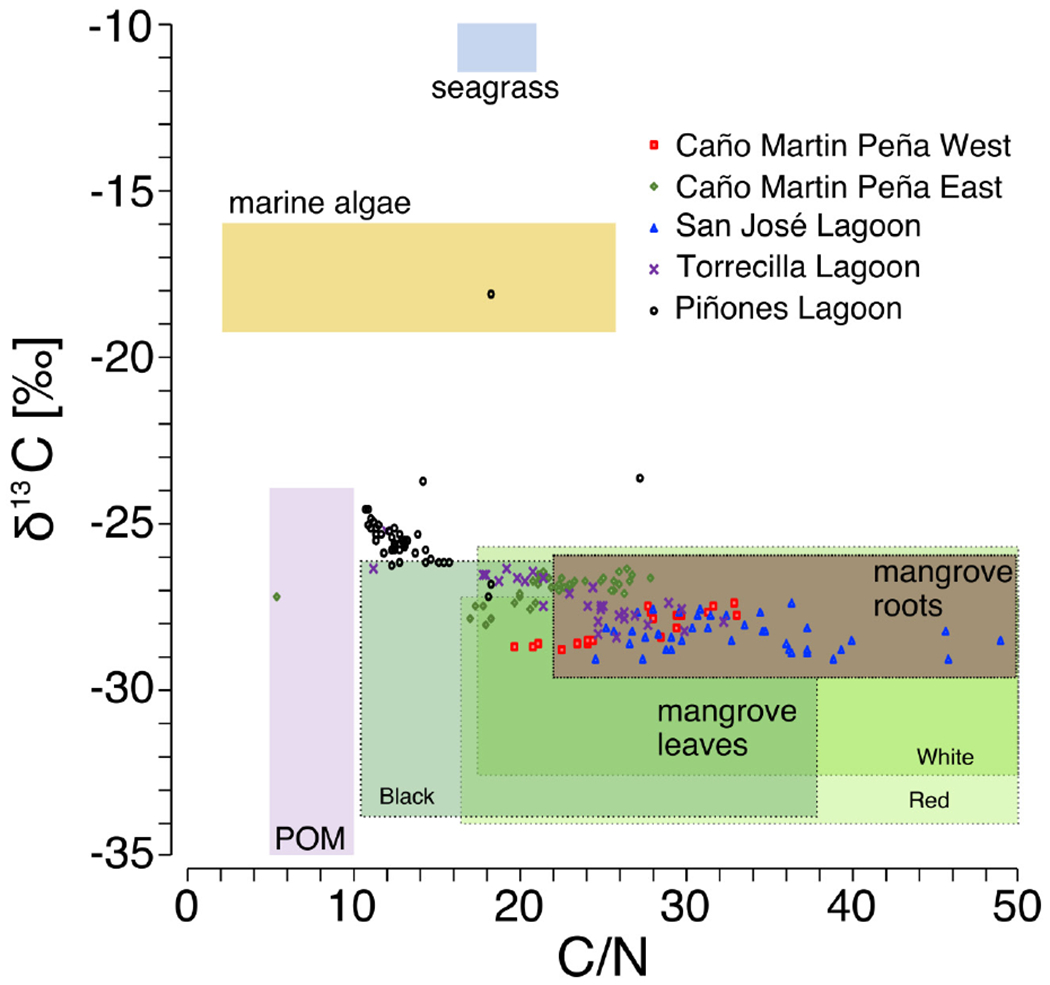
Bi-plot of sediment molar C/N ratio vs sediment C stable isotopes in core sediments relative to potential organic matter sources [source δ^13^C, C/N ratios, and particulate organic matter (POM) based on Khan et al., 2019; Branoff, 2019; Oczkowski et al., unpublished data). Mangrove-derived material was separated into roots and green leaves. Mangrove leaves are further characterized by ranges of C/N ratios and δ^13^C in Red (*Rhizophora mangle* L.), White [*Laguncularia racemosa* (L.) C.F. Gaertn.], and Black [*Avicennia germinans* (L.) L.] species.

Use of stable C isotopes and C/N ratios suggest that most of the SJBE mangrove soils contained mangrove root and leaf sources of organic matter (Figure 4). The leaves of different mangrove species and root matter had overlapping C/N and ´^13^C ranges making it difficult to separate out specific mangrove species as sources in most cases. The Piñones sediment showed evidence that POM supplemented mangrove plant matter in the soil as the C/N ratios and C isotopes were intermediate between POM and mangrove-derived sources. Among the different mangrove sources, Piñones soil most reflected Black [*A. germinans* (L.) L.] mangrove (Figure 4). The site mean δ^13^C at Piñones was significantly more enriched than all other sites in both historic (−25.4‰) and recent (−25.7‰) decades (Table 2 and Supplementary Table 3). In recent decades the site mean δ^13^C of SJ (−28.4‰), Torr (−28.2‰), and MPW (−28.8‰) were significantly less enriched than MPE (−27.3‰) and Piñones (Table 2 and Supplementary Table 3). We observed that the soil ´^13^C was significantly more enriched in historic than recent decades in the Caño Martin Peña cores, La Torrecilla cores, and in Pin2 (Supplementary Table 5).

### Estuary-Wide Mangrove Forest C Storage and Accumulation Rates

**FIGURE 5 |:**
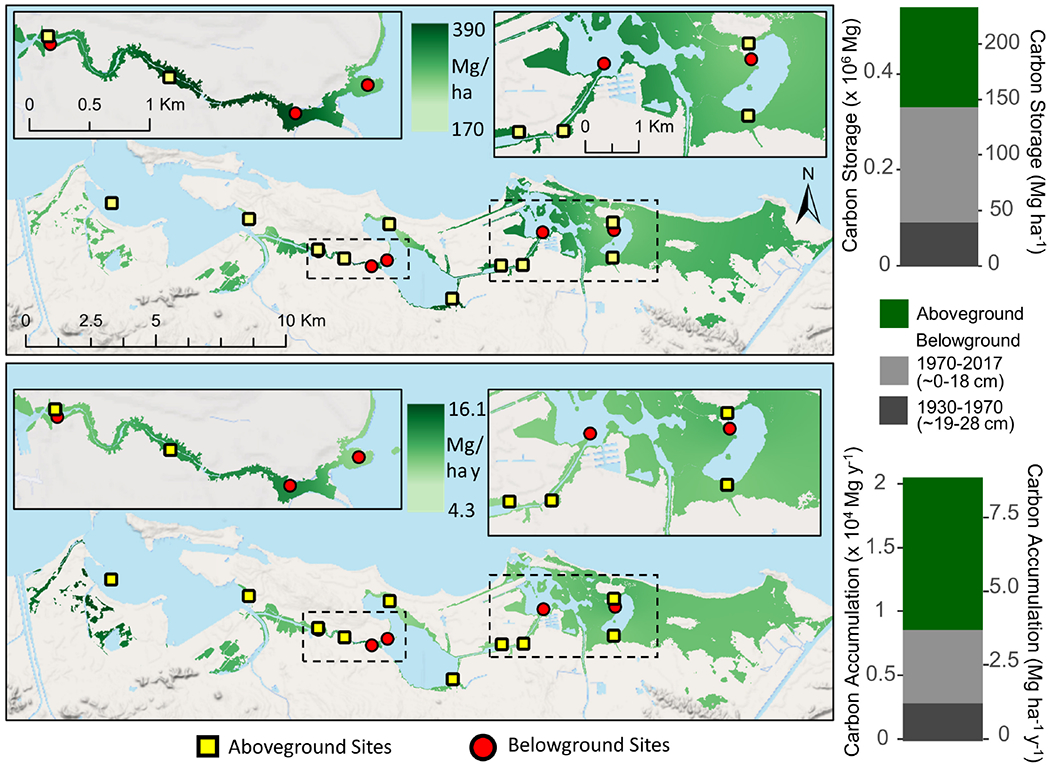
Interpolated mangrove forest carbon storage (top) and accumulation rates (bottom) for both aboveground and belowground soil (<28 cm) components in the San Juan Bay Estuary. Map values represent distance weighted interpolations between known measurements at site locations. Bar graphs represent sums of all mangrove areas across the watershed. Aboveground mangrove sites (Branoff and Martinuzzi, 2020) are denoted by squares and belowground sites (this study) by circles.

Aboveground mangrove C storage was estimated at 208,308 Mg and 90.1 Mg ha^−1^ with a C accumulation rate of 5.2 Mg ha^−1^ y^−1^ for the entire SJBE (Figure 5). Belowground C storage was 240,904 Mg and 104 Mg ha^−1^ in recent decades and 92,013 Mg and 40 Mg ha^−1^ in historic decades (Figure 5). Soil C accumulation rates were estimated at 2.49 Mg ha^−1^ y^−1^ in recent decades and 1.26 Mg ha^−1^ y^−1^ in historic decades.

### Anthropogenic Emission Analysis

Anthropogenic emissions were attributed to a population of 769,000 (~1,850 people km^−2^) and occupied households of 296,589 in the SJBE watershed (U.S. Census Bureau, 2017; Supplementary Table 1b). Annual emissions per household were 1.2 Mg C y^−1^ (equivalent to 4.6 metric tons of CO_2_) plus attributed commercial and industrial electricity use of 2.2 Mg C y^−1^ (equivalent to 7.9 metric tons of CO_2_; Supplementary Table 1c). The per vehicle annual emissions were 1.85 Mg C y^−1^ (equivalent to 6.79 metric tons of CO_2_; Supplementary Table 1c). Residential, commercial, and industrial electricity and vehicular use attributed to total households and persons summed to 1.56 million Mg C y^−1^ (Supplementary Table 1b). We estimated the watershed-wide anthropogenic emission rate at approximately 37.4 Mg C ha^−1^ y^−1^ (watershed area: 41,572 ha). The estuary-wide mangrove forest C sequestration rate (aboveground biomass plus belowground soil: 7.7 Mg C ha^−1^ y^−1^) was 20.6% or about one fifth of the watershed anthropogenic emission rates.

## DISCUSSION

Hydrological alterations (e.g., dredging; canalization; damming), land development, and human activities (e.g., wastewater inputs; filling in) associated with the urbanization of the SJBE in recent decades had direct and indirect effects on soil fertility, hydrology, plant productivity, and sediment deposition. Although there was a distinct urbanization gradient from west to east with the Caño Martin Peña in the west being most urbanized and the Pin the least urbanized, site characteristics such as flushing, landscape setting, and other environmental factors played a role in the C storage and burial rates at the mangrove forest sites. Causes for elevated sediment accretion, C storage, and C accumulation varied among mangrove forest sites in the SJBE.

### Accretion Rates

Mean SAR among the different mangrove forest sites in the SJBE ranged from 1.96–3.76 mm y^−1^ in historic decades and 2.01–5.52 mm y^−1^ in recent decades. Tide gages at San Juan, PR (NOAA station #9755371) and Magueyes Island, PR (NOAA station #9759110) have long-term sea-level rise trends of 2.09 ± 0.37 mm y^−1^ (1962–2020) and 1.90 ± 0.30 mm y^−1^ (1955–2020), indicating mangrove forest accretion rates from the ^210^Pb age models were at or exceeded long-term sea-level rise rates, although the historic period in this study is not covered fully by the water level records. While the SJBE accretion rates in the historic decades were similar to the global median for mangrove forests (2.8 mm y^−1^, 95% confidence interval 1.9–3.9 mm y^−1^; Breithaupt et al., 2012), the accretion rates in recent decades were nearly twice the global median at the most urbanized Caño Martin Peña sites (5.19 mm y^−1^) and least urbanized Pin (5.52 mm y^−1^). Recent SAR and % organic matter at Caño Martin Peña were significantly greater in recent decades compared with historic decades. Such an increase in accretion has been observed in other mangrove forests, coincident with accelerations in sea-level rise (Breithaupt et al., 2020), though, local factors such as anthropogenic wastewater inputs, vegetation cover, and flooding frequency can also affect SAR, especially in peri-urban mangrove systems (Pérez et al., 2018). Elevated accretion rates at the Caño Martin Peña in recent decades may be explained by increased anthropogenic inputs such as raw sewage into the clogged canal; similarly elevated sediment accretion and C accumulation were observed for other urbanized mangrove forests receiving wastewater inputs (Sanders et al., 2014; Pérez et al., 2018, 2020).

### Carbon Storage and Burial Rates

We observed different C storage and burial rates at the western and eastern ends of the urbanized Caño Martin Peña. In the dredged western portion of the canal (MPW), the C burial rates were 58% lower and the C storage 61% lower than the clogged eastern portion (MPE) in recent decades. The site differences in C burial rates and storage between MPW and MPE may be reflective of increased flushing in the MPW in recent times, which might have lowered particulate deposition and C storage. In the eastern clogged end of the Martin Peña, the build-up of autochthonous (i.e., mangrove litterfall and roots) and allochthonous C inputs (e.g., raw sewage and storm runoff) might have contributed to high SAR and C storage. However, the C stable isotope ratios suggest that refractory mangrove litterfall and roots were the apparent C source in the soils (Figure 4). High emissions of carbon dioxide and methane gases measured at the Caño Martin Peña forest and adjoining waters in an earlier report may have been fueled by the more labile allochthonous (e.g., raw sewage) inputs (Martin et al., 2020), diminishing their contributions to long term C storage. The fate of allochthonous inputs and their contributions to C storage needs further study.

Accretion rates and C storage were also high in the low flushed and least urbanized Pin. The Piñones mangrove soils were acidic, had significantly lower porewater pH (4.7), and higher porewater salinities (62–73 ppt) than the other mangrove sites in the SJBE (Martin et al., 2020). Acidic soils are characteristic of organic matter decay and high sulfur oxidation in mature mangrove forests (Alongi et al., 2004; Marchand et al., 2004). Conservation and recycling of nutrients in the Pin would support high productivity and C accumulation in the system. The expansive root system of the multi-species Pin can trap autochthonous C (e.g., litterfall; plant parts) and possibly allochthonous sediments during storm and flooding events (Pérez et al., 2018; Breithaupt et al., 2020). Future studies in the SJBE might be designed to examine the pulse effect of hurricanes and storms on sediment deposition and/or disturbance to mangrove forests (Smith et al., 2009).

Carbon recycling and elevated porewater salinities may in part explain the shift to a more enriched δ^13^C at Piñones, relative to the other SJBE sites. Following the damming of the Río Grande de Loíza in 1953, the Piñones lagoon no longer received water through a series of drainage and navigation canals from the Río Grande de Loíza (Webb and Gómez-Gómez, 1998; Cordero, 2015), resulting in a mostly hydrologically isolated system. Elevated porewater salinities have been reported to significantly decrease intercellular CO_2_ concentrations in mangrove leaves and cause lower C isotopic discrimination and higher transpiration efficiency, resulting in a more enriched δ^13^C (Lin and Sternberg, 1992; Medina and Francisco, 1997). In addition, overland flow of C-4 plant particulates derived from surrounding agricultural lands (e.g., sugarcane) likely contributed to mangrove soils and in part explain a shift to more enriched δ^13^C at Piñones (Spain and Le Feuvre, 1997).

Mangrove soil cores collected from MPE and Torr had the highest within-site variability in DBD, C storage, and C burial rates. Human activities (e.g., filling in with debris; raw sewage inputs) at the clogged MPE site likely increased spatial heterogeneity of the mangrove soils (e.g., particle size, bulk density, and % organic matter). In the 1960s and early 1970s, largescale dredging activities in the northwest inlet connecting Torr with the sea altered the hydrology, distribution and settling of sediment (Oczkowski et al., 2020a). Indirect effects of dredging could also cause high spatial heterogeneity of the mangrove soils in the SJBE.

Our estimate for the estuary-wide mangrove soil C burial rate of 188 g m^−2^ y^−1^ across time periods was similar in magnitude to the global average (174 ± 23 g m^−2^ y^−1^, ±SE; Alongi, 2014) and the global geometric mean (163 g m^−2^ y^−1^, 95% confidence interval 131–202 g m^−2^ y^−1^; Breithaupt et al., 2012). However, the recent decadal time period (1970–2016) did exhibit a greater mean soil C burial rate of 249 g m^−2^ y^−1^, which may reflect some of the disturbances in parts of the SJBE that have been hypothesized to be responsible for extreme C burial rates in other highly impacted sites in Brazil, India, and China (600–1,100 g m^−2^ y^−1^; Alongi, 2014; Sanders et al., 2014; Pérez et al., 2018). In fact, in the highly urbanized and clogged MPE, arguably one of the most disturbed mangrove forests in the SJBE, the recent C burial rate (469 g m^−2^ y^1^) was over twice as great as the global soil C burial estimates.

Brandeis et al. (2014) estimated mangrove forest (above plus belowground biomass) accumulated C at a rate of 11,262 Mg y^−1^ for the SJBE. Dividing by the extant mangrove forest (1,486 ha) reported by Brandeis et al. (2014), would result in a C sequestration rate of 7.6 Mg ha^−1^ y^−1^. In the present study (~5 years later), the sum of mangrove aboveground C accumulation (5.2 Mg ha^−1^ y^−1^) and belowground C burial (2.5 Mg ha^−1^ y^−1^) was 7.7 Mg ha^−1^ y^−^, remarkably similar to the Brandeis et al. (2014) mangrove forest C sequestration rates for the SJBE. Elevated greenhouse gas emissions from SJBE mangrove sediments and adjoining waters, especially in more urbanized areas (e.g., Caño Martin Peña), may partially offset sequestered C in the system (Martin et al., 2020). Rosentreter et al. (2018) determined that global methane emissions from mangrove sediment and waters have the potential to offset C burial rates on average by 20% (using the methane 20-year global warming potential). Therefore, greenhouse gas emissions associated with mangrove systems, which include forest soils, mudflats, and waters, need to be accounted in addition to C accumulation in plant biomass and soils when assessing the importance of mangrove ecosystems in blue carbon inventories. In addition, lateral export of dissolved inorganic and organic C and particulate organic C to oceanic waters may represent a significant C sink, perhaps even greater than the C sequestered in mangrove forest soils, and should also be accounted for in blue C inventories in the future (Maher et al., 2018).

**FIGURE 6 |:**
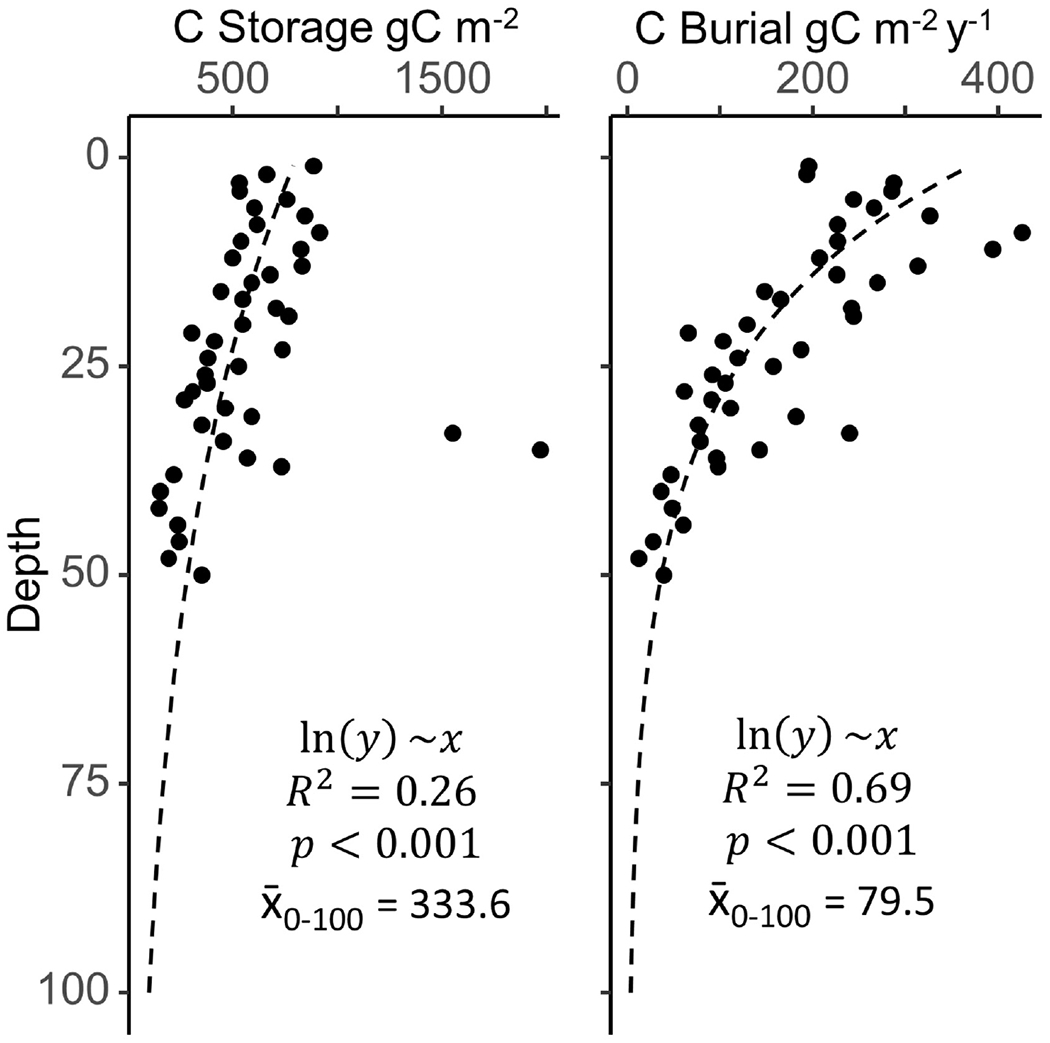
Mean depth profiles for carbon (C) storage and burial across all sites, with the deepest layers approximated by extrapolation of a log transformed linear model (${\bar{\text{x}}}_{0–100}$ is the mean of modeled values from 0 to 100 cm of depth). Filled circles are combined mangrove soil data from nine San Juan Bay Estuary cores. Site specific depth profiles (not shown) varied greatly and may not necessarily follow the estuary-wide generalizations.

When examining the changes in C burial with depth and time (Figure 6), the SJBE in the mid-20th Century exhibited soil C burial rates that more closely resembled the current global average. Other studies have noted changes in hydrology, mangrove coverage, and settling dynamics throughout the various waterbodies of the SJBE over the last half of the twentieth century (Bunch et al., 2000; Martinuzzi et al., 2009; Branoff, 2020b), all of which likely contributed to the observed increases in C burial in recent decades in addition to the constant press of sea-level rise, which has been shown to increase C burial in mangroves (Breithaupt et al., 2020). For mangrove belowground C storage, our estuary-wide estimate of 143 Mg ha^−1^ was less than half of the 382, 419, and 370 Mg ha^−1^ averages for other Caribbean mangroves in the United States, Dominican Republic, and Mexico, respectively (Atwood et al., 2017); however, these averages represent the first 1 m of soil, whereas our SJBE measurements included only the first 18–30 cm. Extrapolating the SJBE C storage values to 1 m (Figure 6) suggested that about 45% of the C was stored in the first 25 cm. Thus, total C storage within the top meter for the whole estuary was estimated at 334 ± 29 Mg ha^−1^ (±SD) which was more similar to other Caribbean mangrove forests and the global soil C stock of 283 ± 193 Mg ha^−1^ (± SD) (Atwood et al., 2017).

### Regional Anthropogenic Carbon Emissions

On an annual basis, the estimated C emissions from watershed households and their vehicles (1.56 million Mg y^−1^) were over 85 times greater than the C sequestered by mangroves (17,713 Mg y^−1^). While the amount of C sequestered by mangrove forests in aboveground biomass and soils was less than that emitted by households each year in the watershed, the C sequestration that mangroves provide is an essential ecosystem service. On an annual basis for every 1.2 Mg C sequestered by mangroves, one household’s electricity use can be offset, and it would take an additional 2.2 Mg C to offset the household’s attributed commercial and industrial electricity use. Similarly, 1.85 Mg C sequestered by mangroves can offset one vehicle’s annual emissions. Thus, the amount of C annually sequestered by SJBE mangroves (17,713 Mg y^−1^) could offset the annual emissions associated with the electricity usage of around 14,800 households or 9,600 vehicles. If we accounted for commercial and industrial activities in addition to household electricity, then the offset would be about 5,200 households. It is not unusual that the emissions from human activity exceed the annual C sequestration from natural processes, as cities are consumptive entities (Odum, 2007). Indeed, in temperate locations, Hall (2011) found that respiration from the burning of fossil fuels exceeded net primary production by 200–700 times depending on the affluence of the neighborhood. At the county level, Balogh et al. (2016) found that respiration exceeded production by 2:1 to 9:1 over the history of a temperate city. Our study focused specifically on coastal mangrove forest C sequestration, and further research might examine how other terrestrial vegetation in the SJBE watershed might offset additional anthropogenic emissions.

## CONCLUSION

Globally, mangroves provide shelter, flood protection, food, and fiber to millions of people in addition to their high C sequestration rates that can mitigate greenhouse gas emissions (Kauffman et al., 2020; Lovelock, 2020). Delivery of these ecosystem services is threatened by land-use conversion, anthropogenic inputs (e.g., urban runoff and sewage), increase in storms, and sea-level rise (Valiela et al., 2001; Bosire et al., 2014). Indeed, land-use change has resulted in substantial reductions in both biomass and soil C stocks (Sasmito et al., 2019), while sea-level rise is predicted to further stress such systems when ecogeomorphic feedbacks do not keep up with rising waters (Saintilan et al., 2020). If mangrove forests persist, they have the potential to help mitigate greenhouse gas emissions, as demonstrated in this study.

In most mangrove forest cores spanning the SJBE, greater soil C storage was measured in recent decades (1970–2016) as compared to historic decades (1930–1970), except for two cores from the SJ and one core from Torr. The mangrove sites in the present study had belowground C burial rates ranging from 88 g m^−2^y^−1^ in the SJ to 469 g m^−2^ y^−1^ in MPE in recent decades. Belowground C burial rates in recent decades were significantly greater than historic decades at the most urbanized Caño Martin Peña and least urbanized Piñones. Not only urbanization, but site-specific flushing patterns, landscape setting, and soil characteristics affected soil C burial rates, and these might be considered when modeling C sequestration in tropical and anthropogenically altered mangrove systems. The watershed anthropogenic CO_2_ emissions (1.56 million Mg C y^−1^) were over 85 times greater than the annual SJBE mangrove forest C storage rates (aboveground biomass plus soils: 17,713 Mg y^−1^). In order to mitigate greenhouse gas emissions in urban, tropical areas, a combination of maintaining healthy mangrove forests, as well as strategies to reduce anthropogenic emissions might be necessary.

## Supplementary Material

SI Zip

## Figures and Tables

**TABLE 1 |:** Categorical factors (landscape setting, flushing, and Urban Index) and the location (latitude/longitude) of the sampling sites.

Site	Mangrove setting	Relative flushing	Urban Index	Core replicates	Latitude/longitude
MPW	Canal (dredged)	Med-high	100	1	N 18 25 59.0808/ W 66 3 30.9348
MPE	Canal (clogged)	Low	88.4	2	N 18 25 42.2184/ W 66 2 21.9408
SJ	Lagoon	Medium	44.8	2	N 18 25 47.676/ W 66 2 3.498
Torr	Lagoon	Med-high	25	2	N 18 26 23.0212/ W 65 58 53.1084
Pin	Forested reserve	Low	1	2	N 18 26 24.972/ W 65 57 23.1912

MPW, Martin Peña West; MPE, Martin Peña East; SJ, San José Lagoon; Torr, La Torrecilla Lagoon; Pin, Piñones Forest.

**TABLE 2 |:** Site comparisons of sediment accretion rate (SAR), mangrove accretion rate (MAR), carbon (C) density, and C stable isotope ratio (δ^13^C) within either the historic or recent time period.

Parameter	Historic (1930–1970) Site differences	Recent (1970-2016) Site differences
SAR	None	SJ, Torr < other sites
MAR	SJ < MPE, MPW, Pin	Pin > MPW, SJ; SJ < all but Torr
C density	Pin < MPE, MPW	MPE > MPW
δ^13^C	Pin1 > MPE, Torr > MPW, SJ	Pin > all; MPE > Torr, SJ, MPW; Torr > MPW

Statistical site differences (P < 0.05) within time periods were based on whether bootstrapped bounds (i.e., 2.5th and 97.5th percentiles) overlapped. Parameter means, lower, and upper confidence bounds were generated on bootstrap runs (1,000 values per core; combined for a total of 2,000 values for sites with two replicates). See Table 1 for site abbreviations.

## Data Availability

The datasets presented in this study can be found in online repositories. The names of the repository/repositories and accession number(s) can be found in the article/Supplementary Material.
