# Supplementary material for: Recent Carbon Storage and Burial Exceed Historic Rates in the San Juan Bay Estuary Peri-Urban Mangrove Forests (Puerto Rico, United States)
Source: Front For Glob Change. Author manuscript; Available in PMC 2022 Feb 2. (PMC8809366; doi:10.3389/ffgc.2021.676691)
Supplement: SI Zip [file NIHMS1771869-supplement-SI_Zip.zip › Data_Sheet_1_Recent Carbon Storage and Burial Exceed Historic Rates in the San Juan Bay Estuary Peri-Urban Mangrove Forests (Puerto Rico, United State.PDF]

Supplementary Table 2. Formulas used to calculate soil carbon (C) storage and burial rates.

| **Parameter** | **Parameter Abbreviation** | **Units** | **Formula** |
|---|---|---|---|
| Mangrove Accretion Rate | MAR | $g\ m^{-2}y^{-1}$ | [a]SAR*[b]DBD |
| Carbon Burial Rate | CBR | $g\ m^{-2}y^{-1}$ | [a]SAR*[b]DBD*%C |
| Carbon Density | C Density | $g\ cm^{-3}$ | %C*[b]DBD |
| Carbon Storage | C Storage | $Mg\ ha^{-1}$ | %C*[b]DBD*[c]Z |

[a] SAR = Sediment Accretion Rate

[b] DBD = Dry Bulk Density

[c] Z = Depth interval defined by specific time period

Supplementary Table 3. Mangrove forest site mean sediment accretion rates (SAR), mangrove accretion rates (MAR), soil carbon (C) density, and carbon stable isotope ratios ($\delta^{13}C$). Parameter means, lower, and upper confidence bounds were generated on bootstrap runs (1000 bootstrap values per core; combined for a total of 2000 values for sites with two replicates). Lower and upper bounds shown in parentheses were 2.5th and 97.5 percentile of 2000 bootstrap means. Sites listed from high to low urbanization index (see Table 1 for site abbreviations).

| | **SAR (mm $y^{-1}$)** | | **MAR (g $m^{-2}$ $y^{-1}$)** | | **C Density (g $cm^{-3}$)** | | **$\delta^{13}C$ (‰)** | |
|---|---|---|---|---|---|---|---|---|
| **Site** | **Historic** | **Recent** | **Historic** | **Recent** | **Historic** | **Recent** | **Historic** | **Recent** |
| MPW | 2.69 | 5.47 | 1176.65 | 2006.84 | 0.052 | 0.036 | -28.01 | -28.79 |
| | (2.20 – 3.20) | (4.17−6.82) | (799−1589) | (1422−2750) | (0.040−0.063) | (0.030−0.043) | (-28.09− -27.97) | (-28.88− -28.69) |
| MPE | 2.66 | 4.91 | 1886.00 | 3704.79 | 0.053 | 0.096 | -26.89 | -27.34 |
| | (1.97−3.35) | (3.93−6.21) | (848−3766) | (1667−5939) | (0.036−0.091) | (0.046−0.164) | (-27.03− -26.73) | (-27.73− -27.08) |
| SJ | 1.96 | 2.02 | 309.77 | 291.81 | 0.049 | 0.043 | -28.53 | -28.44 |
| | (1.39−3.04) | (1.25−3.36) | (154−599) | (128−578) | (0.029−0.074) | (0.028−0.060) | (-29.21− -27.84) | (-28.99− -28.02) |
| Torr | 2.54 | 2.68 | 2512.30 | 1745.00 | 0.072 | 0.094 | -27.46 | -28.15 |
| | (2.00−2.84) | (2.24−3.47) | (560−5521) | (433−3757) | (0.019−0.155) | (0.037−0.177) | (-27.69− -26.89) | (-28.54− -27.79) |
| Pin | 3.76 | 5.52 | 1926.93 | 3770.30 | 0.024 | 0.042 | -25.36 | -25.69 |
| | (2.78−4.98) | (4.51−6.76) | (1306−2784) | (2779−5658) | (0.016−0.033) | (0.028−0.073) | (-26.00− -24.53) | (-25.99− -25.35) |

Supplementary Table 4. Mean mangrove forest core sediment accretion rates (SAR), mangrove accretion rates (MAR), and soil carbon (C) density. Means with lower and upper bounds in parentheses for each core based on 1,000 Bootstrap runs. Lower and upper bounds were 2.5th and 97.5 percentile of 1000 bootstrap means. Site/cores listed from high to low urbanization index (see Table 1 for site abbreviations).

| Site/ Core | SAR (mm $y^{-1)}$ | | MAR (g $m^{-2}$ $y^{-1}$) | | C Density (g $cm^{-3}$) | |
|---|---|---|---|---|---|---|
| | **Historic** | **Recent** | **Historic** | **Recent** | **Historic** | **Recent** |
| MPW | 2.69<br>(2.20 – 3.20) | 5.47<br>(4.17−6.82) | 1176.65<br>(799−1589) | 2006.84<br>(1422−2750) | 0.052<br>(0.040−0.063) | 0.036<br>(0.030−0.043) |
| MPE1 | 3.08<br>(2.67−3.41) | 4.81<br>(4.42−5.19) | 2614.84<br>(1462−3927) | 4848.52<br>(3678−6210) | 0.063<br>(0.037−0.093) | 0.131<br>(0.097−0.169) |
| MPE2 | 2.28<br>(1.89−2.62) | 5.00<br>(3.79−6.50) | 1136.39<br>(815−1506) | 2534.18<br>(1609−3926) | 0.045<br>(0.036−0.056) | 0.061<br>(0.045−0.081) |
| SJ1 | 1.40<br>(1.39−1.41) | 1.28<br>(1.25−1.30) | 215.45<br>(145−293) | 181.46<br>(118−254) | 0.045<br>(0.030−0.060) | 0.039<br>(0.025−0.055) |
| SJ2 | 2.53<br>(2.00−3.16) | 2.76<br>(2.11−3.46) | 393.31<br>(191−658) | 395.27<br>(224−613) | 0.052<br>(0.028−0.077) | 0.046<br>(0.031−0.062) |
| Torr 1 | 2.75<br>(2.65−2.85) | 2.30<br>(2.22−2.37) | 633.69<br>(556−721) | 501.93<br>(427−583) | 0.029<br>(0.018−0.043) | 0.045<br>(0.037−0.055) |
| Torr 2 | 2.33<br>(1.96−2.70) | 3.05<br>(2.54−3.61) | 4415.76<br>(3224−5791) | 3027.19<br>(2191−4035) | 0.116<br>(0.078−0.164) | 0.143<br>(0.107−0.188) |
| Pin 1 | 3.09<br>(2.74−3.40) | 4.82<br>(4.45−5.18) | 1686.58<br>(1269−2096) | 4291.86<br>(2958−5940) | 0.028<br>(0.021−0.035) | 0.053<br>(0.035−0.074) |
| Pin2 | 4.41<br>(3.74−5.07) | 6.20<br>(5.58−7.00) | 2178.37<br>(1547−2895) | 3192.99<br>(2704−3795) | 0.021<br>(0.016−0.027) | 0.031<br>(0.028−0.035) |

Supplementary Table 5. Mean mangrove forest core soil dry bulk density (DBD), percent carbon (%C), and carbon stable isotope ratios ($\delta^{13}C$) estimated for recent and historic time periods. Means with lower and upper bounds in parentheses for each core based on 1,000 Bootstrap runs. Lower and upper bounds were 2.5th and 97.5 percentile of 1000 bootstrap means. Site/cores listed from high to low urbanization index (see Table 1 for site abbreviations); md = missing data.

| Site/ Core | DBD (g cm$^{-3}$) | | %C | | $\delta^{13}C$ (‰) | |
|---|---|---|---|---|---|---|
| | **Historic** | **Recent** | **Historic** | **Recent** | **Historic** | **Recent** |
| MPW | 0.44<br>(0.35−0.53) | 0.37<br>(0.31−0.43) | 11.93<br>(10.26−13.13) | 9.87<br>(9.04−10.74) | -28.01<br>(-28.09− -27.97) | -28.79<br>(-28.88− -28.69) |
| MPE1 | 0.85<br>(0.51−1.25) | 1.02<br>(0.78−1.25) | 7.45<br>(6.84−8.23) | 12.92<br>(10.99−15.02) | -26.79<br>(-26.86− -26.71) | -27.44<br>(−27.78− -27.09) |
| MPE2 | 0.50<br>(0.41−0.61) | 0.50<br>(0.37−0.65) | 8.89<br>(8.78−9.06) | 12.13<br>(10.80−13.55) | -26.99<br>(-27.05− -26.91) | -27.23<br>(-27.41− -27.07) |
| [1]SJ1 | md | md | 29.23<br>(26.98−31.54) | 27.60<br>(26.01−29.30) | -28.15<br>(-28.49− -27.82) | -28.21<br>(-28.46− -27.99) |
| SJ2 | 0.16<br>(0.07−0.25) | 0.14<br>(0.10−0.19) | 33.17<br>(31.89−34.88) | 31.61<br>(30.72−32.54) | -28.93<br>(-29.21− -28.50) | -28.67<br>(-29.03− -28.21) |
| Torr1 | 0.23<br>(0.20−0.26) | 0.22<br>(0.19−0.25) | 12.77<br>(9.20−16.33) | 20.79<br>(18.68−22.43) | -27.63<br>(-27.69− -27.58) | -28.39<br>(-28.54− -28.17) |
| Torr2 | 1.89<br>(1.59−2.18) | 0.99<br>(0.79−1.20) | 6.11<br>(4.36-8.60) | 14.52<br>(11.91−16.85) | -27.27<br>(-27.67− -26.88) | -27.91<br>(-28.03− -27.79) |
| Pin1 | 0.54<br>(0.45−0.64) | 0.91<br>(0.65−1.20) | 5.07<br>(4.24−5.94) | 5.87<br>(5.55−6.24) | -25.69<br>(-26.12− -25.31) | -25.57<br>(-25.81− -25.31) |
| Pin2 | 0.49<br>(0.39−0.60) | 0.52<br>(0.47−0.57) | 4.28<br>(3.57−4.90) | 6.07<br>(5.61−6.54) | -25.02<br>(-25.53− -24.44) | -25.80<br>(-26.02− -25.58) |

[1]DBD values were not determined for SJ1 due to human processing error. The DBD values of SJ2 were used in C storage and burial calculations for both replicates.
